# Wall-Following Behavior for a Disinfection Robot Using Type 1 and Type 2 Fuzzy Logic Systems

**DOI:** 10.3390/s20164445

**Published:** 2020-08-09

**Authors:** M. A. Viraj J. Muthugala, S. M. Bhagya P. Samarakoon, Madan Mohan Rayguru, Balakrishnan Ramalingam, Mohan Rajesh Elara

**Affiliations:** Engineering Product Development Pillar, Singapore University of Technology and Design, 8 Somapah Rd, Singapore 487372, Singapore; bhagya_samarakoon@mymail.sutd.edu.sg (S.M.B.P.S.); madan_rayguru@sutd.edu.sg (M.M.R.); balakrishnan@sutd.edu.sg (B.R.); rajeshelara@sutd.edu.sg (M.R.E.)

**Keywords:** wall following, fuzzy logic systems, wall disinfection, healthcare robotics

## Abstract

Infectious diseases are caused by pathogenic microorganisms, whose transmission can lead to global pandemics like COVID-19. Contact with contaminated surfaces or objects is one of the major channels of spreading infectious diseases among the community. Therefore, the typical contaminable surfaces, such as walls and handrails, should often be cleaned using disinfectants. Nevertheless, safety and efficiency are the major concerns of the utilization of human labor in this process. Thereby, attention has drifted toward developing robotic solutions for the disinfection of contaminable surfaces. A robot intended for disinfecting walls should be capable of following the wall concerned, while maintaining a given distance, to be effective. The ability to operate in an unknown environment while coping with uncertainties is crucial for a wall disinfection robot intended for deployment in public spaces. Therefore, this paper contributes to the state-of-the-art by proposing a novel method of establishing the wall-following behavior for a wall disinfection robot using fuzzy logic. A non-singleton Type 1 Fuzzy Logic System (T1-FLS) and a non-singleton Interval Type 2 Fuzzy Logic System (IT2-FLS) are developed in this regard. The wall-following behavior of the two fuzzy systems was evaluated through simulations by considering heterogeneous wall arrangements. The simulation results validate the real-world applicability of the proposed FLSs for establishing the wall-following behavior for a wall disinfection robot. Furthermore, the statistical outcomes show that the IT2-FLS has significantly superior performance than the T1-FLS in this application.

## 1. Introduction

Emerging infectious diseases affect global socioeconomic stability [1]. These infectious diseases are caused by pathogenic microorganisms such as bacteria, viruses, parasites, or fungi. A majority of diseases can transmit from person to person, and an infectious disease might lead to a pandemic when it spreads globally in an uncontrollable manner. COVID-19, which is a rapidly spreading virus through both direct and indirect contact, can be taken as an example [2,3,4]. Almost all countries are facing difficulties due to this pandemic. The worst aspect of this disease is that a proportion of infected people do not exhibit the symptoms, and their contact areas such as walls, furniture, door handles, and handrails are a threat to other healthy persons. These contaminable surfaces should be frequently disinfected to avoid contact with the virus [5,6].

Conventionally, human laborers conduct the disinfection of public places such as hospitals. They face the risk of exposure to pathogenic organisms when they engage in disinfecting those possible contact areas. The scarcity of human labor is also a major issue during a pandemic situation. Thereby, the conventional disinfection methods that involve extensive human labor face the difficulty of adequately conducting the disinfection of public places. This difficulty hinders the control of the spread of an infectious disease (e.g., COVID-19) among the community. As a solution to this dilemma, robots can be utilized [7,8,9,10,11,12].

The indirect transmission of an infectious disease can be caused by contacting/touching contaminated respiratory droplets in public places. Walls can easily be contaminated with these pathogenic droplets due to closer contact with persons. The highest likely areas are long corridors in hospitals, nursing homes, rapid transit stations, and community centers. Therefore, the walls of these places should often be disinfected to avoid the community spread of the diseases. Robots can be used for spraying disinfectant agents, directing ultraviolet light, or mopping to disinfect the walls [9,11,12,13].

Much work has been conducted on the development of teleoperated robots to provide medical care in outbreaks of an infectious disease, alleviating the risk of workers [14,15,16]. Although these teleoperated robots could be adopted for disinfecting walls, they require the continuous involvement of a human operator. Furthermore, telerobotic control consists of a combination of a master, a slave, and a human operator. Although the individual components are stable in isolation, when combined together, they may be unstable. Therefore, the autonomous abilities of robots are preferred for a robot intended for wall disinfection processes. The critical requirement of an autonomous robot for disinfecting walls is the ability to appropriately follow a wall.

Wall-following behavior for a robot can be achieved by adopting line-following capabilities [17,18]. However, a perceivable line should be marked on the floor near a wall. Hence, the adoption of the line-following methods for the wall-following behavior of a disinfection robot is not a feasible solution for larger environments and general public places. Many research studies have been conducted on navigating a robot in an environment by using Simultaneous Localization and Mapping (SLAM) techniques [19,20]. These methods are capable of navigating a robot in a given trajectory within an environment previously mapped. Therefore, robot navigation methods based on SLAM could be adopted for wall following. However, these methods are prone to fail in situations when a map is not correctly matched with the corresponding actual environment. Moreover, a robot requires an accurate map of an environment, where the robot is deployed. Furthermore, accurately mapping of and navigation in larger environments with fewer features such as corridors in hospitals are problematic. Thereby, there are practical concerns about the usage of SLAM based navigation methods for a robot intended for disinfecting walls in public spaces. In addition to these concerns, autonomous robots with online path planning and decision-making abilities could outperform robots that use offline methods [21,22,23]. Therefore, robots with online path planning abilities are preferred for a wall disinfection robot.

The uncertain handling capacity of fuzzy logic has been exploited by many literature works, on the online decision making intended for wall following. A method based on a Type 1 Fuzzy Logic System (T1-FLS) for safely guiding a wheelchair through wall following was proposed in [24]. The work [25] proposed an Interval Type 2 Fuzzy System (IT2-FLS) to ensure the safety of navigation through wall following. However, the focus of the work [24,25] was ensuring the safety by avoiding the possible collisions with walls, and the methods were not intended for maintaining a given distance from a wall during navigation. Thereby, the cited work could not be adapted for a wall disinfection robot. Methods based on PID controllers for maintaining a given distance from a wall during the wall following have been proposed [26,27]. However, the cited methods were designed for following merely straight walls and require the tuning of the gains for different set-points. This assumption hinders the applicability of the methods to a wall disinfection robot.

The wall-following behavior of different machine learning methods such as neural networks was studied in [28,29,30,31,32], where a majority of the cited work compared the performances of these methods. An open source database that contains the required action of the robot’s corresponding sensor readings for a particular scenario has been used for both training and testing of the algorithms. Moreover, the cited work compared the robot’s action classification ability of the algorithms with corresponding inputs. However, real experiments or simulations with a robot were not conducted for verification other than evaluating the classification ability of the testing dataset. The requirement of training considering all the probable situations for the generalization is the major drawback of these machine learning methods proposed for wall following.

Many studies have been conducted to develop the ability of the wall following in robots using T1-FLSs tuned by methods such as genetic algorithms [33], ant colony optimization [34], bee colony optimization [35,36], and differential evolution [37]. In addition to that, the work [38,39] proposed IT2-FLSs tuned by reinforcement ant optimization and differential evolution, respectively, to establish wall-following behavior. These optimization based fuzzy logic systems are capable of learning the required maneuvering actions to follow walls after training on environments. However, these methods have been designed in such a way that the training is specific for a particular lateral distance from a wall. If the lateral distance from a wall needs to be changed to a new value, the FLSs must be retrained considering the new value before the operation. Moreover, the lateral distance from a wall could not be adjusted per the requirement of the disinfection process before retraining the FLSs. The requirement of training/tuning, considering a large number of possible scenarios for generalization, is the primary concern of these fuzzy logic systems based on optimization techniques.

Much work has been conducted on the development of T1-FLSs based on expert knowledge for developing wall-following ability in mobile robots [40,41,42,43]. Moreover, these FLSs based on expert knowledge have been proposed to resolve the limitation of FLSs tuned through optimization methods. The work [40] compared and contrasted the performance of a PID controller and a T1-FLS designed for establishing wall-following behavior. The main interest of the work was to improve the performance in terms of time taken for following a wall, and the performance of maintaining a desired lateral distance was not evaluated in this work. The T1-FLS proposed in [41] utilizes sonar sensor readings of a mobile robot to realize wall-following behavior. The work [42,43] proposed a behavior based fuzzy controller consisting of three independent T1-FLSs for wall-following. This controller switches between the three T1-FLSs based on the contexts of wall following, straight, left corner, and right corner. The switching between the FLSs is performed based o then conditions evaluated through range sensor readings. Thus, the switching might be rigid in the case of sensor noises. Furthermore, three independent T1-FLSs were required for realizing the wall following using the controller proposed in [42,43]. The T1-FLSs proposed in [40,41,42,43] were capable of realizing the wall-following behavior of a mobile robot, and they could resolve the limitation of FLSs tuned through optimization methods. Nevertheless, the FLSs proposed in [40,41,42,43] were designed considering a specific lateral distance maintained from a wall. Moreover, the membership functions of FLSs must be redesigned to facilitate the alteration of the desired lateral distance. This limitation of the state-of-the-art FLSs designed for wall-following hinders the flexible utilization in a mobile robot intended for wall disinfection. According to [44,45], an IT2-FLS has more ability to cope with uncertainties compared to a T1-FLS in general cases. Nevertheless, a comparison between a T1-FLS and IT2-FLS for establishing the wall-following behavior of a robot has not been conducted.

This paper proposes a novel method for establishing the wall-following behavior for a wall disinfection robot with the focus on enhancing the ability to maintain a given target distance from a wall. The proposed method has the ability to alter the target distance based on user requirements withoutperforming any redesign work. This ability ensures the flexibility of the utilization of the proposed method in a mobile robot intended for wall disinfection over the state-of-the-art FLSs designed for wall following. A non-singleton Type 1 Fuzzy Logic System (T1-FLS) and a non-singleton Interval Type 2 Fuzzy Logic System (IT2-FLS) are individually considered for the proposed method. The ability of the T1-FLS and IT2-FLS to maintain a target distance from a wall during the wall following is systematically evaluated as a core contribution to the state-of-the-art. An overview of the proposed system for wall disinfection is given in Section 2. The FLSs proposed for establishing wall-following behavior are proposed in Section 3. The validation of the proposed FLSs, including a comparison between the T1-FLS and IT2-FLS, is discussed in Section 4. Concluding remarks are given in Section 5.

## 2. System Overview

**Figure 1:**
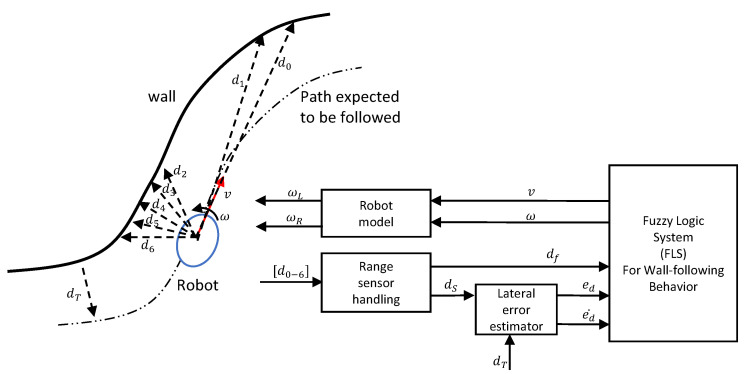
System overview.

An overview of the proposed method is depicted in Figure 1. The base of the wall disinfection robot consists of a differential drive unit, which carries the mechanisms related to the wall disinfection process. It is expected to have either a sprayer gun that sprays a disinfectant agent or ultraviolet light. This disinfection mechanism is directed to the side of the robot (left or right). The effectiveness of a disinfection process depends on the distance between the robot and the wall. For example, if the robot sprays a disinfectant agent, the area coverage and the concentration on the wall surface depend on the distance from the wall. If ultraviolet light is used, the intensity on a wall surface depends on the distance from the wall. Hence, the robot needs to maintain a given lateral target distance (i.e., $d_{T}$) from the wall throughout the process while moving forward.

The robot is intended to be operated in an unknown environment, and the robot is expected to perform maneuvering based on the wall arrangement to follow the wall while maintaining the target distance, $d_{T}$. The robot can perceive the arrangement of the wall through a set of range sensors attached to the robot. A set of Time of Flight (ToF) range sensors is used in this regard. Furthermore, the proposed method has the ability to use sonar sensors since the gap between sensors was considered as 15${}^{\circ }$, which can provide adequate separation for the sonar sensors. The distances measured from the range sensors were considered as $d_{0}$, $d_{1}$, $d_{2}$, $d_{3}$, $d_{4}$, $d_{5}$, and $d_{6}$. It should be noted that the right side of the robot was also fixed with a set of range sensors with a similar arrangement, and those are not depicted here since the interest in this case is the left side. The front and left side regions were defined in such a way that they represent natural human perception. According to [46,47], the front and left regions are dominated from −22.5${}^{\circ }$ to 22.5${}^{\circ }$ (maximum at 0${}^{\circ }$) and from 67.5${}^{\circ }$ to 112.5${}^{\circ }$ (maximum at 90${}^{\circ }$), respectively. A gap of 15${}^{\circ }$ between the sensors was considered for the sonar sensors. Thus, $d_{0}$ and $d_{1}$ were placed at 0${}^{\circ }$ and 15${}^{\circ }$ to cover the front region (a sensor was placed at −15${}^{\circ }$ for the wall following from the right). The sensors $d_{2}$, $d_{3}$, $d_{4}$, $d_{5}$, and $d_{6}$ were fixed at 60${}^{\circ }$, 75${}^{\circ }$, 90${}^{\circ }$, 105${}^{\circ }$, and 120${}^{\circ }$, respectively, to cover the left region. The lateral distance of the robot with the sidewall is defined as $d_{S}$. The closest position within the left region is considered as $d_{S}$ in this regard. Thus, $d_{S}$ in a situation is obtained as $d_{S}=\min\left\{d_{2},d_{3},d_{4},d_{5},d_{6}\right\}$. The range sensor handling module communicates with the sensors and outputs the processed sensor information.

The robot should determine its maneuvering to maintain $d_{S}$ at $d_{T}$ for realizing the wall following and the proper disinfection process. The lateral error between the desired and the actual lateral distance, $e_{d}$, is taken as $e_{d}=d_{T}-d_{S}$. The lateral distance error ($e_{d}$) and the rate of change of $e_{d}$ (i.e., $\dot{e_{d}}$) are fed to the Fuzzy Logic System (FLS) proposed for wall-following behavior. $e_{d}$ and $\dot{e_{d}}$ are determined by the lateral error estimator based on $d_{S}$ and $d_{T}$ ($d_{T}$ is a user-defined constant). The distance from an obstacle at the front (i.e., $d_{f}$) is also fed to the FLS. The range sensors $d_{0}$ and $d_{1}$ are the corresponding sensors in the case of the wall following from the left. The closest distance within the frontal region is considered to ensure safety. Thus, $d_{f}$ is defined as $d_{f}=\min\left\{d_{0},d_{1}\right\}$. The FLS determines the reference/desired linear velocity (*v*) and angular velocity (ω) of the robot required to properly follow the wall based on its inputs. A Type 1 Fuzzy Logic System (T1-FLS) and an Interval Type 2 Fuzzy Logic System (IT2-FLS) were considered for the FLS proposed for wall-following behavior. The linear velocity (*v*) and angular velocity (ω) determined by the FLS are fed into the robot model, where it determines the required/reference angular velocities of the left and right wheels of the robot ($\omega_{L}$ and $\omega_{R}$). In other words, the output of the FLS decides the set-point inputs for the lower level PID controllers acting on DC motors attached to the wheels. For the sake of brevity and without any loss of generality, the desired/reference terms are dropped in the remainder of the paper. The variation of the angular velocities of the wheels makes the robot follow the wall as expected.

## 3. Fuzzy Logic System for Wall-Following Behavior

A Fuzzy Logic System (FLS) can map the input space with the output space of a control problem based on a set of linguistic rules [48,49]. Fuzzy logic can be considered as a reliable modeling method that can model any complex system or process without knowing the exact underlying dynamics [50,51,52]. In the case of wall-following behavior, the exact dynamics of the robot and an environment is not certain. Nevertheless, the required control actions for wall-following behavior could be explained using linguistic expressions based on expert knowledge [53]. Furthermore, the sensory information retrieved from the range sensors of the robot is imprecise due to sensor noise. On the contrary, fuzzy logic has proven to be effective at inferring control actions while coping with imprecise sensor information [54,55]. In addition to that, fuzzy logic has often been used for the navigation of robots in unknown environments [53,56]. Therefore, fuzzy logic was used to establish the wall-following behavior for the robot. Two fuzzy logic systems, a Type 1 Fuzzy Logic System (T1-FLS) and an Interval Type 2 Fuzzy Logic System (IT2-FLS), were individually considered to realize the required goals of wall-following behavior.

### 3.1. Type 1 Fuzzy Logic System

**Figure 2:**
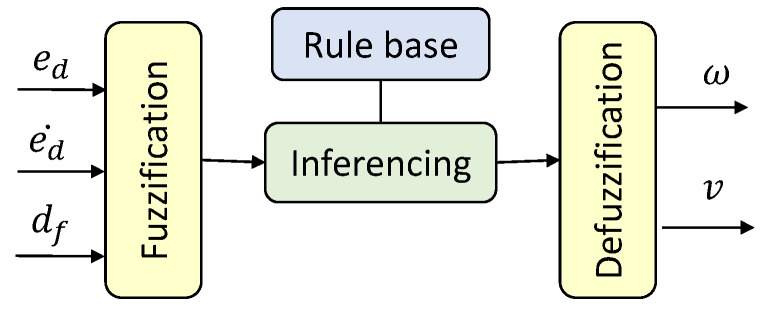
Architecture of the Type 1 Fuzzy Logic System (T1-FLS).

**Figure 3:**
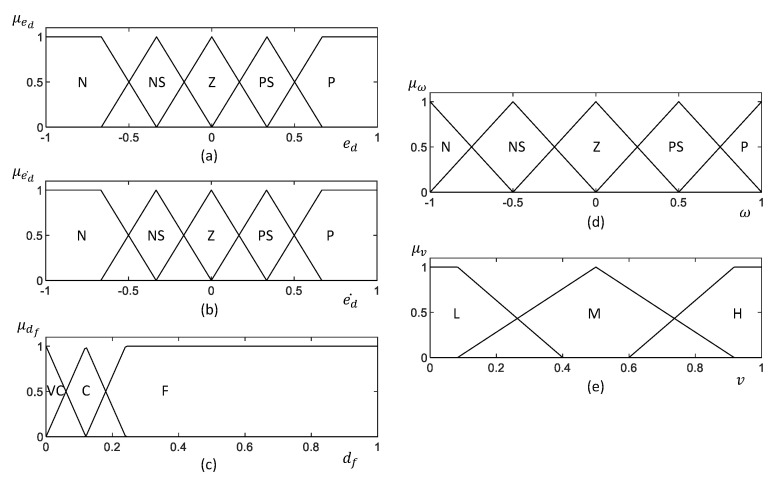
Membership functions of the T1-FLS. (**a**–**c**) input membership function for $e_{d}$, $\dot{e_{d}}$, and $d_{f}$, respectively. (**d**,**e**) Output membership function for ω and *v*, respectively. Fuzzy labels are defined as N: “Negative,” NS: “Negative Small,” Z: “Zero,” PS: “Positive Small,” P: “Positive,” VC: “Very Close,” C: “Close,” F: “Far,” L: “Low,” M: “Medium,” H: “High.” The universe of discourse of the membership functions is defined in the normalized scale, and they should be adapted based on the hardware capabilities of the robot. It should be noted that human expert knowledge was utilized to prune the membership functions.

The architecture of the Type 1 Fuzzy Logic System (T1-FLS) proposed for establishing the wall-following behavior of a mobile robot is depicted in Figure 2. The inputs of the FLS are the lateral distance from the wall error (i.e., $e_{d}$), the rate of change of lateral distance from the wall error (i.e., $\dot{e_{d}}$), and the distance from the front obstacle (i.e., $d_{f}$). The outputs of the T1-FLS are the angular velocity (i.e., ω) and linear velocity (i.e., *v*) of the robot. The inputs are fuzzified in the fuzzification layer by using the input membership functions given in Figure 3a–c for $e_{d}$, $\dot{e_{d}}$, and $d_{f}$, respectively. Non-singleton fuzzy sets are defined for the input membership functions since they can cope more effectively with uncertainties than the singleton counterparts [57]. The corresponding fuzzified values are defined as $\mu_{e_{d}}\left(e_{d}\right)$, $\mu_{\dot{e_{d}}}\left(\dot{e_{d}}\right)$, and $\mu_{d_{f}}\left(d_{f}\right)$.

The input fuzzy sets are mapped to output fuzzy sets during the inferencing with the aid of a set of linguistic rules stored in the rule base. The rule base of the T1-FLS is given in Table 1. The rule base is defined based on human expert knowledge. Moreover, the necessary control actions are inferred in this stage. The firing strength of the ith rule, $R_{i}$, can be inferred as given in (1) by considering the min and max operators as the t-norm and t-conorm, respectively.

(1) $$R_{i}=\min\left\{\mu_{e_{d_{i}}}\left(e_{d}\right),\mu_{\dot{e_{d_{i}}}}\left(\dot{e_{d}}\right),\mu_{d_{f_{i}}}\left(d_{f}\right)\right\}$$

The corresponding output fuzzy sets are clipped by the respective firing strength of the ith rule as given in (2) by considering the Mamdani implication, where $\mu_{\omega_{i}^{\prime }}\left(\omega \right)$ and $\mu_{v_{i}^{\prime }}\left(v\right)$ are the inferred ith consequents of the outputs ω and *v*, respectively. The fuzzy consequents of all the rules are aggregated into a single fuzzy set for each output ($\mu_{\omega^{\prime }}$ for *w* and $\mu_{v^{\prime }}$ for *v*) as given in (3) by considering the fuzzy max operator for the aggregation, where *N* is the number of rules.

(2) $$\begin{array}{c} \mu_{\omega_{i}^{\prime }}\left(\omega \right)=\min\left\{R_{i},\mu_{\omega_{i}}\left(\omega \right)\right\}\\ \mu_{v_{i}^{\prime }}\left(v\right)=\min\left\{R_{i},\mu_{v_{i}}\left(v\right)\right\} \end{array}$$

(3) $$\begin{array}{c} \mu_{\omega^{\prime }}\left(\omega \right)=\max\left\{\mu_{\omega_{1}^{\prime }}\left(\omega \right),\mu_{\omega_{2}^{\prime }}\left(\omega \right),..,\mu_{\omega_{i}^{\prime }}\left(\omega \right),..,\mu_{\omega_{N}^{\prime }}\left(\omega \right)\right\}\\ \mu_{v^{\prime }}\left(v\right)=\max\left\{\mu_{v_{1}^{\prime }}\left(v\right),\mu_{v_{2}^{\prime }}\left(v\right),..,\mu_{v_{i}^{\prime }}\left(v\right),..,\mu_{v_{N}^{\prime }}\left(v\right)\right\} \end{array}$$

**Figure 4:**
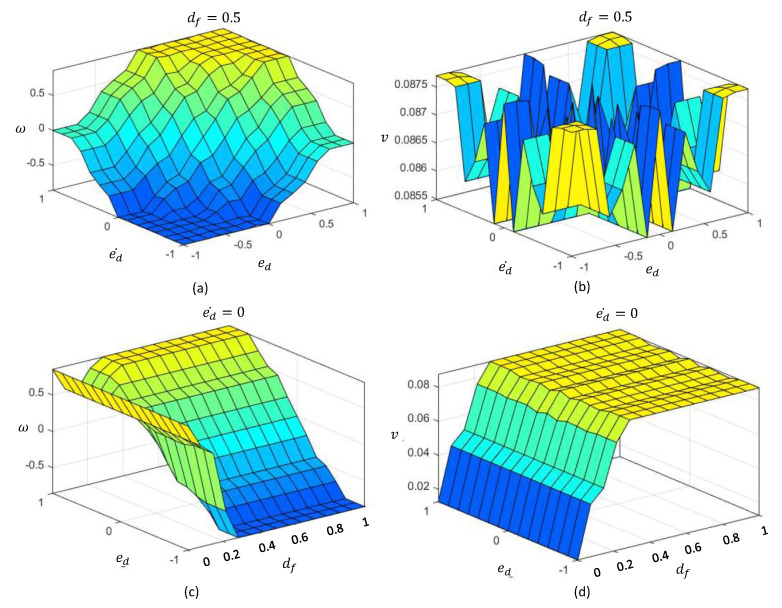
Decision surface of the T1-FLS. (**a**) Variation of *w* with $e_{d}$ and $\dot{e_{d}}$, when $d_{f}=0.5$. (**b**) Variation of *v* with $e_{d}$ and $\dot{e_{d}}$, when $d_{f}=0.5$. (**c**) Variation of *w* with $e_{d}$ and $d_{f}$, when $\dot{e_{d}}=0$. (**d**) Variation of *v* with $e_{d}$ and $d_{f}$, when $\dot{e_{d}}=0$.

The aggregated fuzzy consequents are defuzzified in the defuzzification layer to obtain the deterministic control actions to control angular velocity, ω*, and linear velocity, v*. The defuzzified crisp outputs can be obtained as in (4) based on the center of the area method. The expected decision surface of the proposed T1-FLS, which visualizes the variation of the two outputs in accordance with the inputs, is given in Figure 4.

(4) $$\begin{array}{c} \omega *=\frac{\int \omega \mu_{\omega^{\prime }}\left(\omega \right)d\omega }{\int \mu_{\omega^{\prime }}\left(\omega \right)d\omega }\\ v*=\frac{\int v\mu_{v^{\prime }}\left(v\right)dv}{\int \mu_{v^{\prime }}\left(v\right)dv} \end{array}$$

### 3.2. Interval Type 2 Fuzzy Logic System

Type 2 Fuzzy Logic Systems (T2-FLSs) have proven to be more effective at coping with uncertainties with respect to the T1-FLSs since they allow extra degrees of freedom for modeling an uncertain situation or process [44,45]. Interval Type 2 Fuzzy Logic Systems (IT2-FLSs) are a subtype within the general T2-FLSs. An IT2-FLS has a simpler structure and requires lower computational power than a general T2-FLS, which led researchers to utilize IT2-FLS in practical applications [58,59]. Nevertheless, IT2-FLSs are more complex and require high computational power while providing better performance than that of T1-FLSs.

**Figure 5:**
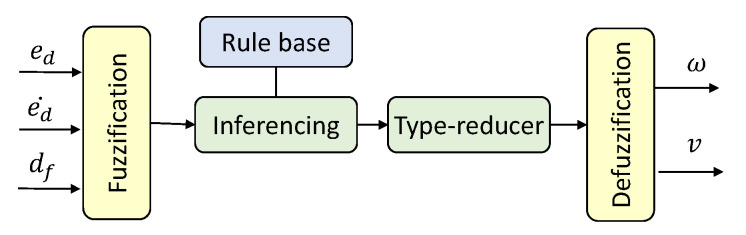
Architecture of the Interval Type 2 Fuzzy Logic System (IT2-FLS).

**Figure 6:**
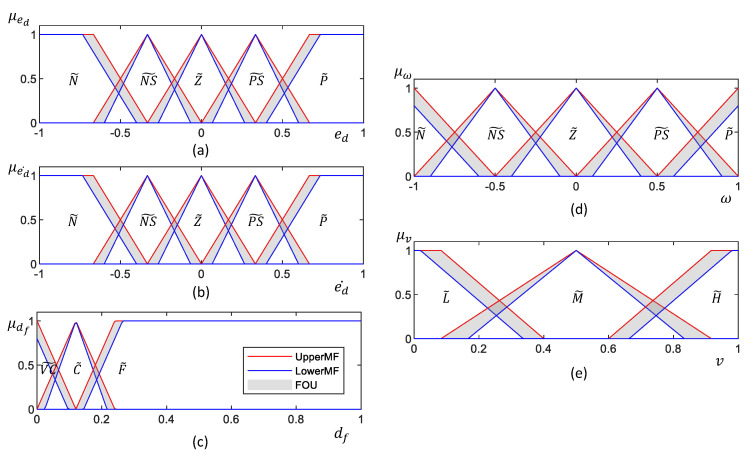
Membership functions of the IT2-FLS. (**a**–**c**) Input membership function for $e_{d}$, $\dot{e_{d}}$, and $d_{f}$, respectively. (**d**,**e**) Output membership function for ω and *v*, respectively. The upper membership function and lower membership function of each fuzzy set are annotated as UpperMF and LowerMF. The Footprint Of Uncertainty (FOU) is represented by the shaded area. Fuzzy labels are defined as N: “Negative,” NS: “Negative Small,” Z: “Zero,” PS: “Positive Small,” P: “Positive,” VC: “Very Close,” C: “Close,” F: “Far,” L: “Low,” M: “Medium,” H: “High.” The universe of discourse of the membership functions is defined in the normalized scale, and they should be adapted based on the hardware capabilities of the robot. It should be noted that the membership functions were pruned based on human expert knowledge.

The architecture of the proposed IT2-FLS for establishing wall-following behavior is depicted in Figure 5. The set of inputs and outputs used here is similar to the proposed T1-FLS. The inputs are initially fuzzified in the fuzzification layer with the aid of the input membership functions shown in Figure 6. Nevertheless, the fuzzification process moderately differs from the T1-FLS. A fuzzy set of an IT2-FLS has a Footprint Of Uncertainty (FOU), which is bounded by two fuzzy sets in a T1-FLS, the Lower Membership Function (LMF) and the Upper Membership Function (UMF) (see Figure 6). Thereby, fuzzification of the input by an IT2-FLS leads to a range instead of a crisp value for the activation degree. This range can be obtained by fuzzifying an input considering the LMF and UMF separately. Based on this formulation, the corresponding fuzzified inputs of the proposed IT2-FLS can be defined as $\left[{\underline{\mu }}_{e_{d}}\left(e_{d}\right),{\bar{\mu }}_{e_{d}}\left(e_{d}\right)\right]$, $\left[{\underline{\mu }}_{\dot{e_{d}}}\left(\dot{e_{d}}\right),{\bar{\mu }}_{\dot{e_{d}}}\left(\dot{e_{d}}\right)\right]$, and $\left[{\underline{\mu }}_{d_{f}}\left(d_{f}\right),{\bar{\mu }}_{d_{f}}\left(d_{f}\right)\right]$ for the inputs $e_{d}$, $\dot{e_{d}}$, and $d_{f}$, respectively, where “_” and “$\;\bar{}\;$” denote resultants from the LMF and UMF, respectively. Moreover, two crisp values are obtained per fuzzy set during the fuzzification of an input.

Similar to the T1-FLS, the proposed IT2-FLS uses a rule base for the inferencing. The same rule base defined for T1-FLS given in Table 1 is used in the proposed IT2-FLS. In contrast, two firing strengths are received for a rule considering LMFs and UMFs. The firing strength of LMFs, ${\underline{R}}_{i}$, and UMFs, ${\bar{R}}_{i}$, of the ith rule can be obtained as in (5). Min and max fuzzy operators are considered as t-norm and t-conorm operators. Moreover, each rule has a range for the firing strength instead of a crisp firing strength.

(5) $$\begin{array}{c} {\underline{R}}_{i}=\min\left\{{\underline{\mu }}_{e_{d_{i}}}\left(e_{d}\right),{\underline{\mu }}_{\dot{e_{d_{i}}}}\left(\dot{e_{d}}\right),{\underline{\mu }}_{d_{f_{i}}}\left(d_{f}\right)\right\}\\ {\bar{R}}_{i}=\min\left\{{\bar{\mu }}_{e_{d_{i}}}\left(e_{d}\right),{\bar{\mu }}_{\dot{e_{d_{i}}}}\left(\dot{e_{d}}\right),{\bar{\mu }}_{d_{f_{i}}}\left(d_{f}\right)\right\}\\ {\tilde{R}}_{i}\equiv \left[{\underline{R}}_{i},{\bar{R}}_{i}\right]\;\;\;\;\;\;\;\;\;\;\;\;\;\;\;\; \end{array}$$

The corresponding LMFs and UMFs of the outputs are clipped by the range of firing strength based on the Mamdani implication. This implication yields the fuzzy consequents ${\tilde{\mu }}_{\omega_{i}^{\prime }}\left(\omega \right)$ and ${\tilde{\mu }}_{v_{i}^{\prime }}\left(v\right)$ for the outputs ω and *v*, respectively, for the ith rule as given in (6). Moreover, each rule produces an interval Type 2 fuzzy set as a consequent for an output. It should be notated that ${\underline{\mu }}_{\omega_{i}^{\prime }}\left(\omega \right)$ and ${\bar{\mu }}_{\omega_{i}^{\prime }}\left(\omega \right)$ represent the LMF and UMF of ${\tilde{\mu }}_{\omega_{i}^{\prime }}\left(\omega \right)$, which is an interval Type 2 fuzzy set. The same notation is also used for ${\tilde{\mu }}_{v_{i}^{\prime }}\left(v\right)$.

(6) $$\begin{array}{c} {\underline{\mu }}_{\omega_{i}^{\prime }}\left(\omega \right)=\min\left\{{\underline{R}}_{i},{\underline{\mu }}_{\omega_{i}}\left(\omega \right)\right\}\\ {\bar{\mu }}_{\omega_{i}^{\prime }}\left(\omega \right)=\min\left\{{\bar{R}}_{i},{\bar{\mu }}_{\omega_{i}}\left(\omega \right)\right\}\\ {\underline{\mu }}_{v_{i}^{\prime }}\left(v\right)=\min\left\{{\underline{R}}_{i},{\underline{\mu }}_{v_{i}}\left(v\right)\right\}\\ {\bar{\mu }}_{v_{i}^{\prime }}\left(v\right)=\min\left\{{\bar{R}}_{i},{\bar{\mu }}_{v_{i}}\left(v\right)\right\} \end{array}$$

Similar to the T1-FLS, the inferred LMFs and UMFs of inferred output fuzzy sets are aggregated considering the fuzzy max operator. The aggregated output fuzzy sets, ${\tilde{\mu }}_{\omega^{\prime }}\left(\omega \right)$ and ${\tilde{\mu }}_{v^{\prime }}\left(v\right)$, can be obtained as in (7) for the outputs ω and *v*. However, the aggregated fuzzy sets are interval Type 2 fuzzy sets, and hence, the fuzzy sets cannot be directly defuzzified the same as Type 1 fuzzy sets. To facilitate the defuzzification, a step called type reduction, which reduces an interval Type 2 fuzzy set to an interval-valued Type 1 fuzzy set, is performed in the type reducer. The Karnik–Mendel [60] algorithm is used for the type reduction in the proposed FLS. An interval Type 1 fuzzy set resulting from the type reduction has a range with a lower limit and an upper limit. Suppose $c_{L}^{\omega }$ and $c_{R}^{\omega }$ are the lower and upper limits of the output ω and $c_{L}^{v}$ and $c_{R}^{v}$ are the lower and upper limits of the output *v*. These lower and upper limits can be estimated as given in (8) and (9). Here, $N^{\omega }$ and $N^{v}$ are the number of samples taken across the universe of discourse of the outputs ω and *v*, respectively. $L^{\omega }$ and $R^{\omega }$ are switch points corresponding to the output ω, and $L^{v}$ and $R^{v}$ are switch points relevant to the output *v*. These switch points are iteratively estimated using the Karnik–Mendel method [60].

(7) $$\begin{array}{c} {\tilde{\mu }}_{\omega^{\prime }}\left(\omega \right)=\max\left\{{\tilde{\mu }}_{\omega_{1}^{\prime }}\left(\omega \right),{\tilde{\mu }}_{\omega_{2}^{\prime }}\left(\omega \right),..,{\tilde{\mu }}_{\omega_{i}^{\prime }}\left(\omega \right),..,{\tilde{\mu }}_{\omega_{N}^{\prime }}\left(\omega \right)\right\}\\ {\tilde{\mu }}_{v^{\prime }}\left(v\right)=\max\left\{{\tilde{\mu }}_{v_{1}^{\prime }}\left(v\right),{\tilde{\mu }}_{v_{2}^{\prime }}\left(v\right),..,{\tilde{\mu }}_{v_{i}^{\prime }}\left(v\right),..,{\tilde{\mu }}_{v_{N}^{\prime }}\left(v\right)\right\} \end{array}$$

(8) $$\begin{array}{c} c_{L}^{\omega }=\frac{\sum_{j=1}^{L^{\omega }}\omega_{j}{\bar{\mu }}_{\omega^{\prime }}\left(\omega_{j}\right)+\sum_{j=L^{\omega }+1}^{N^{\omega }}\omega_{j}{\underline{\mu }}_{\omega^{\prime }}\left(\omega_{j}\right)}{\sum_{j=1}^{L^{\omega }}{\bar{\mu }}_{\omega^{\prime }}\left(\omega_{j}\right)+\sum_{j=L^{\omega }+1}^{N^{\omega }}{\underline{\mu }}_{\omega^{\prime }}\left(\omega_{j}\right)}\\ c_{R}^{\omega }=\frac{\sum_{j=1}^{R^{\omega }}\omega_{j}{\underline{\mu }}_{\omega^{\prime }}\left(\omega_{j}\right)+\sum_{j=R^{\omega }+1}^{N^{\omega }}\omega_{j}{\bar{\mu }}_{\omega^{\prime }}\left(\omega_{j}\right)}{\sum_{j=1}^{R^{\omega }}{\underline{\mu }}_{\omega^{\prime }}\left(\omega_{j}\right)+\sum_{j=R^{\omega }+1}^{N^{\omega }}{\bar{\mu }}_{\omega^{\prime }}\left(\omega_{j}\right)} \end{array}$$

(9) $$\begin{array}{c} c_{L}^{v}=\frac{\sum_{j=1}^{L^{v}}v_{j}{\bar{\mu }}_{v^{\prime }}\left(v_{j}\right)+\sum_{j=L^{v}+1}^{N^{v}}v_{j}{\underline{\mu }}_{v^{\prime }}\left(v_{j}\right)}{\sum_{j=1}^{L^{v}}{\bar{\mu }}_{v^{\prime }}\left(v_{j}\right)+\sum_{j=L^{v}+1}^{N^{v}}{\underline{\mu }}_{v^{\prime }}\left(v_{j}\right)}\\ c_{R}^{\omega }=\frac{\sum_{j=1}^{R^{v}}v_{j}{\underline{\mu }}_{v^{\prime }}\left(v_{j}\right)+\sum_{j=R^{v}+1}^{N^{v}}v_{j}{\bar{\mu }}_{v^{\prime }}\left(v_{j}\right)}{\sum_{j=1}^{R^{v}}{\underline{\mu }}_{v^{\prime }}\left(v_{j}\right)+\sum_{j=R^{v}+1}^{N^{v}}{\bar{\mu }}_{v^{\prime }}\left(v_{j}\right)} \end{array}$$

**Figure 7:**
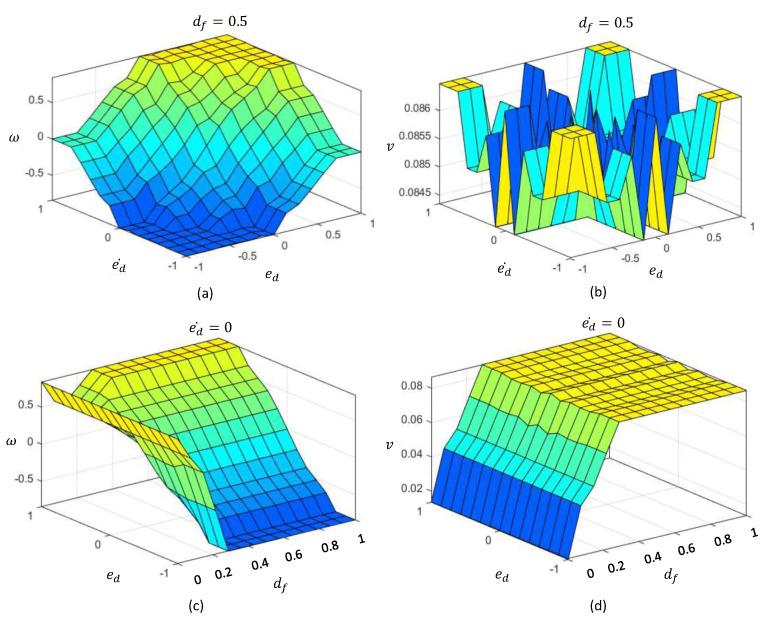
Decision surfaces of the IT2-FLS. (**a**) Variation of *w* with $e_{d}$ and $\dot{e_{d}}$, when $d_{f}=0.5$. (**b**) Variation of *v* with $e_{d}$ and $\dot{e_{d}}$, when $d_{f}=0.5$. (**c**) Variation of *w* with $e_{d}$ and $d_{f}$, when $\dot{e_{d}}=0$. (**d**) Variation of *v* with $e_{d}$ and $d_{f}$, when $\dot{e_{d}}=0$.

The final defuzzified outputs can be obtained as in (10). The expected decision surface of the proposed IT2-FLS, which visualizes the variation of the two outputs in accordance with the inputs, is given in Figure 7. The decision surfaces of the proposed IT2-FLS show higher non-linearity than those of the proposed T1-FLS.

(10) $$\begin{array}{c} \omega *=\frac{c_{L}^{\omega }+c_{R}^{\omega }}{2}\\ v*=\frac{c_{L}^{v}+c_{R}^{v}}{2} \end{array}$$

## 4. Results and Discussion

### 4.1. Validation Setup

A simulation was conducted using MATLAB Simulink to validate the characteristics and performance of wall-following behavior established for a robot by using the proposed Type 1 Fuzzy Logic System (T1-FLS) and Interval Type 2 Fuzzy Logic System (IT2-FLS). The validation process was conducted in two phases. The characteristics of the proposed FLSs were analyzed in the first phase by considering heterogeneous situations of typical walls. In the second phase, the average absolute displacement error of the paths (defined as $E=\int_{0}^{T}\left|d_{e}\right|/T$ where *T* is the total run time in a test case) followed by the robot with the T1-FLS and the IT2-FLS was compared by simulation trails on complex heterogeneous wall arrangements. The performance of the proposed method with different lateral distances to be maintained by the robot from a wall (i.e., with different $d_{T}$) was examined in the third phase of the validation. The inputs, $d_{e}$ and $\dot{d_{e}}$, and outputs, ω and *v*, of the FLSs were directly taken/fed from/to the robot without taking a calibration gain. However, a calibration gain of 0.2 was used for the input, $d_{f}$, of the FLSs. Besides, the inputs were truncated to the respective universe of discourse of the input space. The maximum ranges of the distance sensors were assumed as 4 m. The resolution of the range sensors was considered as 1 cm for the simulation. The lateral distance to be maintained by the robot from the wall (i.e., $d_{T}$) was configured as 0.5 m for the first and second phases of the validation. The maximum linear velocity of the robot was assumed as 1 m/s. This value was chosen based on the requirements of the disinfection process, as well as the typical parameters of mobile robot bases.

### 4.2. The Characteristics of Wall-Following Behavior

**Figure 8:**
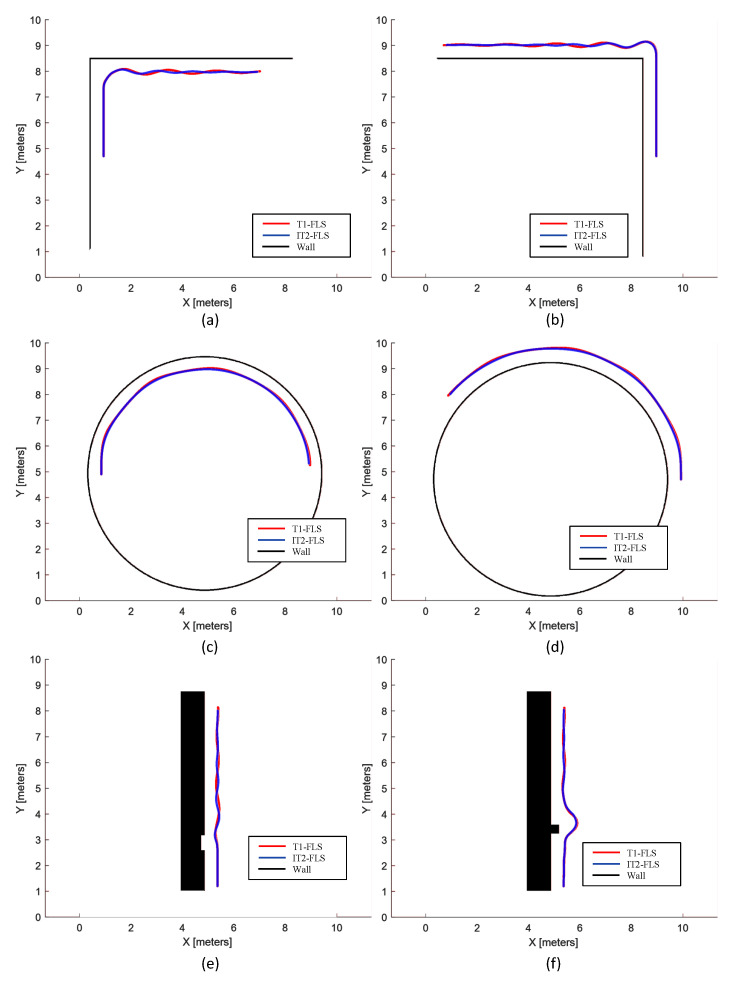
Wall-following behavior of the robot with the T1-FLS and the IT2-FLS during the test cases. (**a**) An inner wall segment of a right-angle corner. (**b**) An outer wall segment of a right-angle corner. (**c**) An inner surface of a curved wall. (**d**) An outer surface of a curved wall. (**e**) A wall segment with a small negative dent. (**f**) A wall segment with a small positive dent.

**Figure 9:**
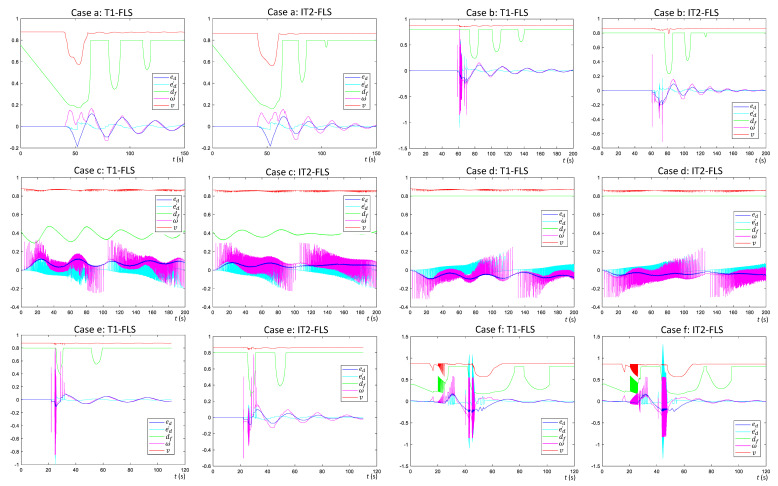
The variation of the inputs and outputs of the T1-FLS and the IT2-FLS during the test cases given in Figure 8.

In this phase of the validation, heterogeneous scenarios where the robot encounters typical walls such as corners and curves were considered individually to analyze the characteristics of the proposed FLSs for wall-following behavior. The arrangement of walls in the test cases of the first phase of the validation is shown in Figure 8 along with the resulting trails of the robot with the T1-FLS and the IT2-FLS. The variations of the parameters of the FLSs corresponding to the test cases are given in Figure 9. It should be noted that for each test case, initially, the robot was placed in such a way that the robot’s heading was parallel to the tangent to the wall surface, and $d_{S}=d_{T}$.

A wall arrangement with a right-angle corner, as shown in Figure 8a, was considered for the case “a”. The robot was placed on the inner side of a right-angle corner of a wall. A robot could often encounter this sort of wall segment when applying the disinfectant. The traced path of the robot for both FLSs are annotated on the map. The corresponding variations of the inputs and the outputs of the FLSs are given in Figure 9. Initially, $d_{f}$ was 0.75, where it activated only the “Far” fuzzy set of the input for $d_{f}$. As a result of sole activation of the “Far” set of $d_{f}$, the robot velocity, *v*, was at its maximum, and the robot tried to minimize $e_{d}$ by varying ω. When the robot moved toward the corner, $d_{f}$ decreased gradually. When $d_{f}$ dropped to a level that activated the “Close” set, *v* started to decrease, and ω started to increase in the clockwise direction to turn the robot to the right. This behavior helped the robot follow the right-angle turn of the wall at the corner. However, after completion of the turn at the corner, the robot had a small $e_{d}$. The robot managed to steady $e_{d}$ after a few oscillations, which were small in magnitude (less than 0.1 m). The peak magnitudes and the number of the oscillations of the IT2-FLS were lower than those of the T1-FLS. The average absolute displacement error of the robot’s path (*E*) was 0.04 m and 0.02 m for the case of the T1-FLS and the IT2-FLS, respectively, indicating a slightly better performance for the IT2-FLS than the T1-FLS in this case. The results of the case “a” verified that both proposed FLSs for wall-following behavior were capable of coping with a situation where there could be right-angle corners in inner wall segments.

A situation where a robot moves along the outer surface of a wall segment that has a right-angle corner was considered for the case “b” (see Figure 9b). Both configurations of the robot followed the first leg of the wall without having a lateral error ($e_{d}=0$). In this case, the robot could not identify the corner by considering $d_{f}$, similar to the case “a”. Nevertheless, soon after the robot passes the first leg of the wall, it perceives the outward turn of the wall through $e_{d}$. Therefore, both the FLSs took necessary action to turn the robot to the left by raising ω in the anti-clockwise direction. After that, both FLSs were capable of following the second wall segment as expected. Similar to the case “a”, both FLSs were able to maintain a lower *E* (0.03 m and 0.02 m for the T1-FLS and the IT2-FLS, respectively). The IT2-FLS showed better performance than that of the T1-FLS. The characteristics identified from this case validated the ability of the proposed FLSs to follow the outer surface of a right-angle wall corner.

The characteristics of the proposed FLSs in following a curve wall from the inner side were evaluated in the case “d” shown in Figure 8c. The robot was successful at following the curved wall in both configurations (i.e., with the T1-FLS and with the IT2-FLS) while having a lower *E* (0.07 m and 0.05 m for the T1-FLS and the IT2-FLS, respectively). In the case “d”, the proposed FLSs were tested when the robot was following the same curved wall from outside (see Figure 8d). The proposed FLSs were capable of following the wall surface while maintaining the average lateral error, *E*, within an acceptable range (0.06 m and 0.04 m for the T1-FLS and the IT2-FLS, respectively) for the disinfection process. The IT2-FLS exhibited a steady path with lower peaks of $e_{d}$ compared to the T1-FLS, yielding a superior performance. The characteristics of the proposed FLSs identified from the cases “c” and “d” validated the ability of the proposed systems to cope with curved walls when following the inner and outer sides.

A scenario where there was a small negative dent in the wall surface (see Figure 8e) was considered for the case “e”. This kind of negative dent can be commonly found in corridors due to the inclusion of doors. In contrast, a situation where there was a small positive dent in a wall (see Figure 8f) was considered for the case “f”. This sort of situation is commonly created by structures like pillars and beams. Thereby, the analysis of the characteristics of the proposed FLSs during such situations is crucial. Initially, the robot smoothly followed the wall, and when it encountered a dent, a change in the trajectory was triggered by the FLSs. This triggering allowed the robot to safely pass the positive dent in the case “f”. The robot with the IT2-FLS showed smoother paths than the T1-FLS, where *E* was 0.02 m (Case “e”) and 0.05 m (Case “f”) for the IT2-FLS and 0.03 m (Case “e”) and 0.03 m (Case “f”) for the T1-FLS. The identified characteristics validated the ability of the proposed FLSs to cope with possible negative and positive dents in walls when performing the wall following.

In all the test cases, both proposed FLSs were effective at successfully maintaining the distance from the wall within an acceptable range during the wall following. Moreover, the proposed FLSs could be utilized for establishing the wall-following behavior of a robot intended for disinfecting walls. However, better performance could be observed from the IT2-FLS compared to the TL-FLS. Therefore, a performance comparison of the two FLSs was conducted in the second phase of the validation (given in Section 4.3) by considering complex wall arrangements.

### 4.3. Performance Comparison of Wall-Following Behavior

**Figure 10:**
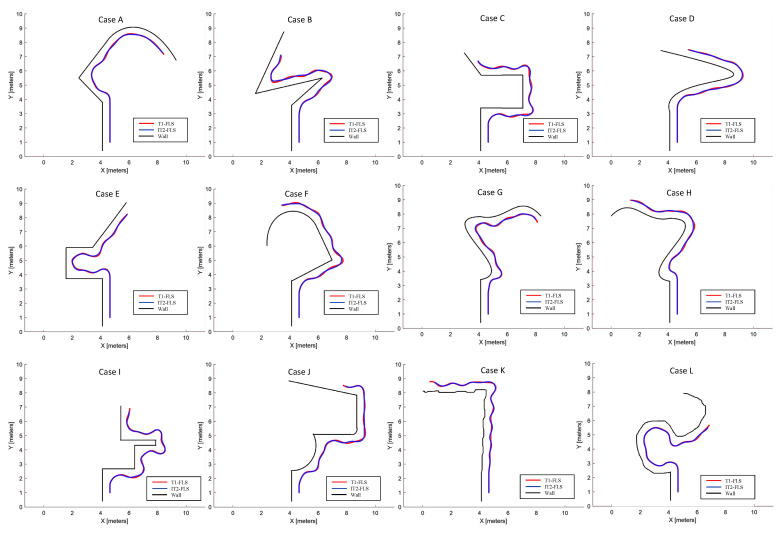
The traced path of the robot during the second phase of the validation. Cases A to L represent the test scenarios.

In this phase of the validation, the performance of the proposed FLSs was compared by considering complex wall arrangements. The wall arrangements considered in this regard are shown in Figure 10. Similar to the first phase of the validation, initially in these cases, the robot was placed in such a way that the robot’s heading was parallel to the tangent to the wall surface, and $d_{S}=d_{T}$. A total of 12 heterogeneous wall arrangements were considered, and the robot (with the T1-FLS and with the IT2-FLS) was allowed to follow the wall for 200 s in each case. The trajectories of the robot were captured. The traced paths of the robot during these test cases were also annotated. A video compiled from the simulations of this phase can be found as a supplementary multimedia attachment.

**Figure 11:**
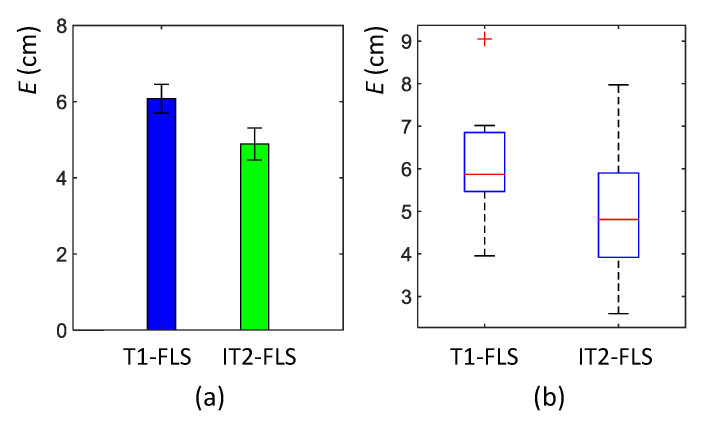
(**a**) Mean of average lateral error, *E*, during the test cases of the second phase of validation. The error bars are drawn to represent the standard error. (**b**) The corresponding box plot of *E*. The box plot has the usual standard notation; box: interquartile range, horizontal line: median, whiskers: minimum and maximum, and plus sign: outliers.

According to the obtained results of these 12 cases, both proposed FLSs for wall-following behavior were capable of successfully following the wall arrangement of the test cases. However, the IT1-FLS generated smoother paths for the robot than the T1-FLS. The average lateral error of each case (i.e., *E*) was calculated for both FLSs to analyze this performance variation quantitatively. In all cases except Case “k”, the robot with the IT2-FLS had a lower *E* than that of T1-FLS. The mean variation of *E* obtained for the test cases is given in Figure 11a with error bars drawn to represent the standard error. In addition to that, the corresponding box plots of *E* are given in Figure 11b to provide insights into the data distribution. The mean of *E* for the T1-FLS was 6.1 cm, with a standard deviation of 1.5. In contrast, the mean of *E* for the IT2-FLS was 4.9 cm, with a standard deviation of 1.3. A paired t-test was conducted to evaluate the statistical significance between the two means. According to the test outcomes, the IT2-FLS had a significantly lower mean for *E* compared to that of the T1-FLS ($t_{\left(11\right)}=7.19$, p=0.00). Furthermore, this error reduction was large according to the effect size calculated based on Cohen’s d (Cohen’s d = 0.86; Cohen’s d > 0.8 is considered as a large effect size [61]). Therefore, it can be concluded that the IT2-FLS had significantly superior performance than the T1-FLS in wall-following behavior, and the performance difference was noteworthy.

### 4.4. Performance of Wall-Following with Different Lateral Distances

**Figure 12:**
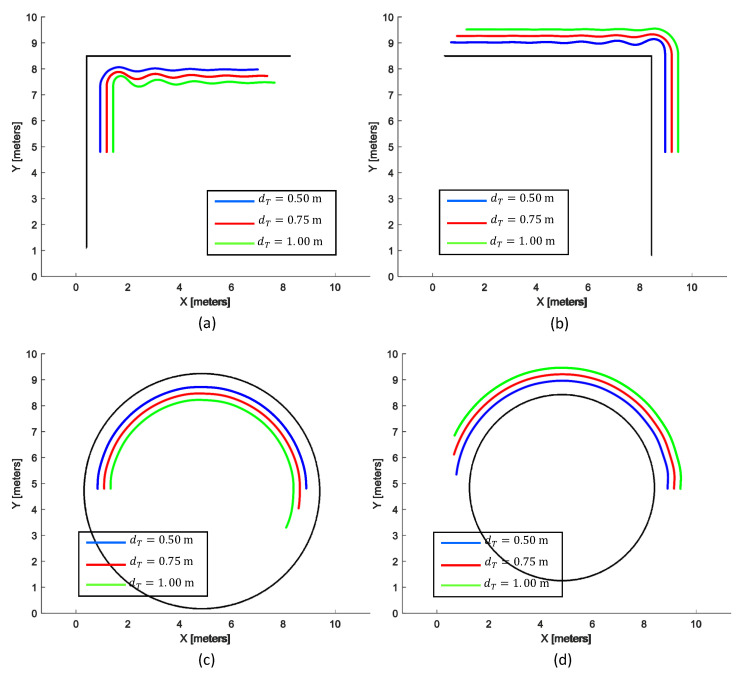
Wall-following behavior of the IT2-FLS with different settings of $d_{T}$. (**a**) An inner wall segment of a right-angle corner. (**b**) An outer wall segment of a right-angle corner. (**c**) An inner surface of a curved wall. (**d**) An outer surface of a curved wall.

In the third phase of the validation, the wall-following ability of the proposed method was examined by configuring different lateral distances (i.e., $d_{T}$). Only the proposed IT2-FLS was considered in this regard since superior performance was observed. Environments similar to the first four cases of the first phase of the experiment were considered. Figure 12 shows the resulting trails of the robot with different configurations of $d_{T}$. Three settings of $d_{T}$, $d_{T}$ = 0.5 m, 0.75 m, and 1.0 m were considered for the analysis.

According to the traced path of the robot, the robot was capable of adequately following the wall segment of each environment for all the configurations of $d_{T}$. The average lateral errors (i.e., *E*) of the cases of $d_{T}$ = 0.5 m, 0.75 m, and 1.0 m were observed as 3.66 cm (SD = 0.41), 3.53 cm (SD = 0.63), and 3.87 cm (SD = 1.41), respectively. Moreover, an adequate level of performance could be observed even though $d_{T}$ was altered. This behavior implied that the proposed FLS was generalized for a finite range of $d_{T}$ instead of a specific value. Therefore, it can be concluded that the lateral distance of a wall-following task can be altered per the user requirements without the requirement of any redesign work of the proposed FLS.

### 4.5. Discussion

The characteristics of the proposed FLSs were evaluated in the first phase of the validation. The test cases of this phase consisted of typical heterogeneous situations that could be encountered by a robot during the wall following. The path followed by the robot and the variation of the inputs and outputs of the FLSs were analyzed in this regard. The wall-following performance of the proposed FLSs was analyzed and compared in the second phase of the validation by considering complex wall arrangements. Based on the outcomes of all the test cases (including both the first and second phase), it can be concluded that both proposed FLSs were effectively capable of coping with typical situations expected during wall-following behavior. Moreover, the proposed FLSs were suitable for establishing the wall-following behavior for a robot deployed for wall disinfection tasks.

In the second phase of validation, the performances of the proposed FLSs in maintaining the desired distance from a wall were compared. Complex wall arrangements that could be encountered by a disinfection robot were considered in this regard. This consideration supported the generalization of the outcomes. In addition to that, statistical analysis was conducted to ascertain the generalizability. The statistical outcomes confirmed that the proposed IT2-FLS had significantly superior performance to the proposed T1-FLS for wall-following behavior. Moreover, the proposed IT1-FLS was more suitable for a wall disinfection robot since it could follow a given wall while maintaining the distance from the wall more accurately.

The evaluation and comparison of the performance were conducted based on the distance maintaining performance, and the other performance indicators such as time and energy usage were not considered. However, the ability to maintain a given distance from a wall while wall following is the most crucial aspect for a wall disinfection robot. Hence, the consideration of distance maintaining ability for the performance evaluation and comparison was justifiable. The consideration of other performance indicators such as time and energy for the analysis is proposed for future work.

The state-of-the-art wall-following methods based on FLSs tuned by optimization techniques (e.g., [33,34,35,36,37,38,39]) require training before utilization. Furthermore, these methods are not designed in a way that the system is generalized to the lateral distance. Hence, retraining is required to alter the lateral distance to be maintained from a wall. On the other hand, the FLSs designed for wall following based on expert knowledge do not require training. However, state-of-the-art FLSs proposed in this regard (e.g., [40,41,42,43]) have been designed considering a specific lateral distance to be maintained from a wall. Moreover, the state-of-the-art FLSs designed for wall-following behavior require redesigning of the FLSs in the case of altering the distance. In contrast, the FLSs proposed in this paper do not require such redesigning since the FLSs consider the parameters related to the error of lateral distance. Moreover, the membership functions of the FLSs are independent of the lateral distance. The flexibility of altering the lateral distance to be maintained in a wall-following task is useful for a robot intended for wall disinfecting since the lateral distance has to be altered per the requirement of the disinfecting process.

Most of the time, the proposed method for establishing the wall-following behavior is capable of maintaining a given distance from a wall during a wall-following task. However, the deviations of the distance from the desired distance could be observed in concave regions. The method proposed for wall-following behavior is expected to be utilized in a wall disinfection robot that uses either a disinfectant spraying mechanism or ultraviolet light directed at the wall. Thereby, occasional minor deviations of the distance from the wall surface would not hinder the effectiveness of a robot-aided disinfection task. However, fusing a method to adapt the parameters of the spraying mechanism or ultraviolet light, such as intensity, based on the present distance from a wall, would benefit from further improving the effectiveness of the method proposed in this paper. For example, if the distance from the wall was higher than the desired one, the spaying mechanism could be adapted to have slightly more pressure. Further exploration in this direction is proposed for future work.

If there is an object beside a wall, the robot considers such an object as a part of the wall. If there is an object beside the wall, contaminating the wall itself is less likely since the wall segment itself would not be contactable by people. However, the likelihood of contaminating such objects would be high. Therefore, considering an object beside a wall as a part of the wall is desirable and would not hinder the effectiveness.

The implications discussed earlier were derived based on the characteristics and performance of the proposed method identified based on the simulation results. The simulations were conducted by considering realistic parameters and models for the robot and environments. Thereby, the results of the simulation would not greatly deviate from real-world experiments. Moreover, the implications concluded in the paper would be applicable to real-world scenarios. The robot’s effective workspace is planar due to the following reasons. The robot perceives only planner information of the environment from the range sensors placed surrounding the robot in the same plane. Furthermore, the robot’s actions are limited to a planer workspace since only the navigation of the robot is considered in the paper. Therefore, a 2D simulation environment is adequate for evaluating the behavior and the performance of the work proposed in the paper. Real-world experiments with a robot equipped with a disinfection mechanism are expected to be conducted in the next phase of the work.

## 5. Conclusions

Robot-aided solutions are demanded to disinfect possibly contaminated surfaces to reduce the spread of infectious diseases. A wall disinfection robot should be capable of following a given wall while maintaining a specified distance from the wall. The ability to maintain the specified distance from the wall is crucial for the effective sterilization of the wall surface since the intensity of the disinfectant agent applied on the wall depends on the distance. The disinfectant agent can be either a sprayable liquid, a gas, or ultraviolet light directed at the wall. Therefore, this paper proposed a novel method to establish the wall-following behavior for a disinfection robot.

The proposed method was designed in such a way that it enables a robot to follow a wall of an unknown environment by online path planning based on the range sensor information. Fuzzy logic was used for establishing the wall-following behavior based on the range sensor inputs. A Type 1 Fuzzy Logic System (T1-FLS) and an Interval Type 2 Fuzzy Logic System (IT2-FLS) were proposed in this regard. The FLSs were designed in such a way that they were generalized from the lateral distance to be maintained from a wall. Moreover, users can alter the lateral distance per the requirements without performing any redesign work of the system. The FLSs take the information perceived from range sensors as inputs and determine the linear velocity and the angular velocity of the robot required to follow a wall successfully.

Simulations were conducted to evaluate the characteristics and performance of the proposed FLSs. Heterogeneous test cases that consisted of typical wall arrangements were considered in the simulation. According to the simulation results, both proposed FLSs (i.e., T1-FLS and IT2-FLS) were capable of adequately following walls as expected for a wall disinfection robot. A superior performance could be observed from the IT2-FLS compared to that of the T1-FLS, and this performance difference was large and statistically significant. The real-world applicability of the proposed method was verified from the simulations, and it is expected that real-world trials will be conducted in the next phase of the work.

## Figures and Tables

**Table 1:** Rule base of the FLSs.

	$\dot{e_{d}}$ \$e_{d}$	N	NS	Z	PS	P
$d_{f}$ = F	N	ω = P *v* = H	ω = P *v* = H	ω = P *v* = H	ω = PS *v* = H	ω = Z *v* = H
	NS	ω = P *v* = H	ω = P *v* = H	ω = PS *v* = H	ω = Z *v* = H	ω = NS *v* = H
	Z	ω = P *v* = H	ω = PS *v* = H	ω = Z *v* = H	ω = NS *v* = H	ω = N *v* = H
	PS	ω = PS *v* = H	ω = Z *v* = H	ω = NS *v* = H	ω = N *v* = H	ω = N *v* = H
	P	ω = Z *v* = H	ω = NS *v* = H	ω = N *v* = H	ω = N *v* = H	ω = N *v* = H
$d_{f}$ = C	ω = NS *v* = M					
$d_{f}$ = VC	ω = N *v* = S					

## Supplementary Materials

The following are available at https://www.mdpi.com/1424-8220/20/16/4445/s1.
